# Genomics in Personalized Nutrition: Can You “Eat for Your Genes”?

**DOI:** 10.3390/nu12103118

**Published:** 2020-10-13

**Authors:** Veronica A. Mullins, William Bresette, Laurel Johnstone, Brian Hallmark, Floyd H. Chilton

**Affiliations:** 1 Department of Nutritional Sciences, University of Arizona, Tucson, AZ 85719, USA; vamullins@arizona.edu (V.A.M.); bresette@email.arizona.edu (W.B.); 2 The BIO5 Institute, University of Arizona, Tucson, AZ 85719, USA; laureljo@arizona.edu (L.J.); bhallmark@statlab.bio5.org (B.H.)

**Keywords:** precision nutrition, nutrigenomics, nutrigenetics, personalized, diet, genetics, nutrients

## Abstract

Genome-wide single nucleotide polymorphism (SNP) data are now quickly and inexpensively acquired, raising the prospect of creating personalized dietary recommendations based on an individual’s genetic variability at multiple SNPs. However, relatively little is known about most specific gene–diet interactions, and many molecular and clinical phenotypes of interest (e.g., body mass index [BMI]) involve multiple genes. In this review, we discuss direct to consumer genetic testing (DTC-GT) and the current potential for precision nutrition based on an individual’s genetic data. We review important issues such as dietary exposure and genetic architecture addressing the concepts of penetrance, pleiotropy, epistasis, polygenicity, and epigenetics. More specifically, we discuss how they complicate using genotypic data to predict phenotypes as well as response to dietary interventions. Then, several examples (including caffeine sensitivity, alcohol dependence, non-alcoholic fatty liver disease, obesity/appetite, cardiovascular, Alzheimer’s disease, folate metabolism, long-chain fatty acid biosynthesis, and vitamin D metabolism) are provided illustrating how genotypic information could be used to inform nutritional recommendations. We conclude by examining ethical considerations and practical applications for using genetic information to inform dietary choices and the future role genetics may play in adopting changes beyond population-wide healthy eating guidelines.

## 1. Introduction

Science in the 20th century yielded a basic understanding of the key macro and micro nutritional requirements for most humans. This resulted in a one-size-fits-all approach, exemplified by plans such as MyPlate and the Food Guide Pyramid, which have been impactful in reducing malnutrition and diseases resulting from nutrient deficiencies [1]. Precision nutrition, sometimes called personalized nutrition, nutrigenetics, or nutritional genetics, is the opposite—individuals receive diets tailored to their personal biology. Studies of global human genomic variation have demonstrated dramatic population-based differences in allele frequencies of common single nucleotide polymorphisms (SNPs) that influence the expression of genes responsible for the metabolism of some of the most common nutrients consumed by humans. In addition, evolutionary studies reveal that humans genetically adapted to their ancestral diets and local environments, as well as genetically drifted apart, giving rise to observed global patterns of sequence variation [2]. Consequently, individuals in large modern populations with diverse genetic ancestries such as the US may have a wide range of metabolic responses to the same food or diet, calling into question the one-size-fits-all dietary approach.

Diet-based genetic variation developed initially in Africa and continued as modern humans migrated out of Africa and across the globe over the past 100,000 years. Natural selection in response to new climates and food sources resulted in population- or region-specific genetic variation [3]. For example, the ability to digest lactose as an adult is much more common among Northern Europeans than East Asians or Africans [4,5]. In addition to these evolutionary studies, genome-wide association studies (GWAS) have discovered many genetic variants associated with specific nutrition-related traits including nutrient absorption, lipid metabolism, nutrient utilization, and fat accumulation that in turn can result in gene–diet interactions and human diseases. Together, these findings raise the critical question of whether dietary recommendations could be tailored to individuals based on genetic variation and how significant the impact of precision nutrition could be in contrast to conventional recommendations. Given the early nature of this science, it is not possible to adequately evaluate the overall effect of precision versus conventional nutrition. It is therefore our objective in this review to highlight both important examples in which genetic information can be helpful or vital in making nutritional recommendations and other examples in which it has limited value.

A growing number of companies now offer direct-to-consumer, genetically-based nutritional testing (DTC-GT) and advice [6]. The rapid growth of this industry is a testament to the fact that large numbers of consumers yearn for the purported benefits of “gene-based diets”. However, precision nutrition is at a very early stage and in most cases lacks sufficient science to be implemented, especially given the complexity of genetic alterations, and their effects, as well as a lack of knowledge of the dietary exposure necessary to induce a detrimental gene–diet interaction. In this review, we discuss the prospects for precision nutrition using genetic information in the context of genome biology, human genetics, and dietary exposure.

## 2. Genomic Architecture

**Figure 1:**
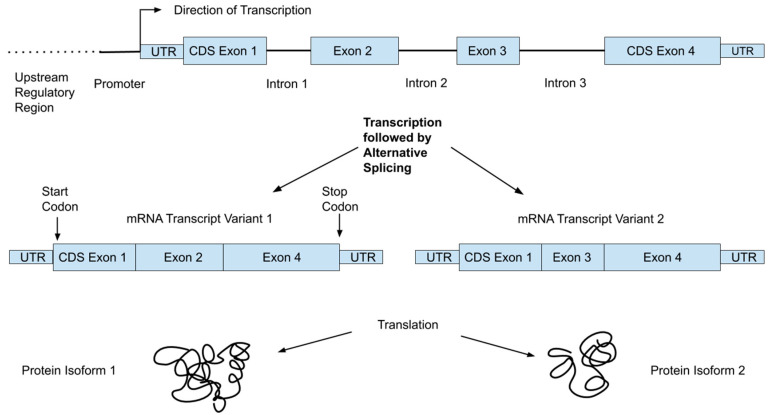
Basic architecture of a gene showing exons (eventually become the mature mRNA transcript), introns (removed during transcription), coding sequence regions within exons (CDS), and untranslated portions of exons (UTR).

The human genome consists of over 3 billion DNA base-pairs organized into chromosomes and present in the nuclei of most of our cells in two copies: one from each parent. It encodes the proteins our bodies need in linear units of information called genes, of which there are about 21,000 [7]. Genes occupy only a small fraction (<1%) of the genome; the rest includes “regulatory machinery”—regions that are important for controlling the transcription of various genes—as well as repetitive regions and large regions with unknown function(s) [7]. Transcriptional machinery “reads” the DNA code and produces mRNA. This mRNA then moves to ribosomes, where it interacts with translational machinery to link amino acids into the encoded proteins. Figure 1 shows an example of the organization of a typical gene, which consists of multiple regions referred to as exons and introns. While both are initially transcribed, spliceosomes remove the intronic regions, such that only exons are present in the mature mRNA transcripts. In addition, a single gene can produce multiple transcripts that each only contain certain exons, allowing single genes to encode multiple protein isoforms [8]. For reference, we provide a glossary of common genetic terms with simple explanations in Table 1.

### 2.1. Genetic Variation

Despite being phenotypically quite diverse, humans are genetically mostly the same, with two individuals differing at <1% of their genomes on average (https://www.ncbi.nlm.nih.gov/books/NBK20363/). There are multiple ways two genomes can differ, and the simplest and most widely studied type of genetic variation is single base pair differences known as single nucleotide polymorphisms (SNPs). Other types of variation include insertions and deletions of short DNA fragments (INDELs); copy number variants (CNV), where a given gene is present in multiple copies and that number varies by individual; and structural variants (SVs), where larger genomic rearrangements exist. Although our knowledge of INDELs, CNVs, and SVs is growing, most of the nutritional genomics research to date has focused on SNPs, and those are the central focus for this review as well. The Genome Reference Consortium (https://www.ncbi.nlm.nih.gov/grc) helps to maintain a regularly updated human reference genome, and variation herein is described in relation to that reference.

Since the human genome was first sequenced in 2003, several large international projects such as HapMap [9], the Human Genome Diversity Panel [10], and the 1000 Genomes Project [11] have worked to sequence the genomes of thousands of individuals from around the world, and have created large catalogues of human genetic variation. We now know that SNPs exist about every 1000 bp, and over 300 million SNPs have been found to date [12]. The popular dbSNP database (https://www.ncbi.nlm.nih.gov/snp/), hosted by the National Center for Biotechnology Information, currently contains information on over 100 million SNPs (National Center for Biotechnology Information, U.S. National Library of Medicine).

### 2.2. From Genotype to Phenotype

Although the central dogma of molecular biology is that the progression from DNA to RNA to protein is straightforward, multiple changes can occur along that process that alter the expression of genes, and in turn the influence the effect of any genetic variant. While a detailed discussion of gene expression or epigenetics is beyond the scope of this review, an important point is that individual variants may not be expressed equally in all individuals. Determining SNP genotypes is straightforward but understanding the complex molecular and metabolic network of events impacted by an individual variant is far more difficult. Some SNP sites have known functions or associations with diseases or other phenotypic characteristics, including metabolism of dietary components and nutritional deficiencies, but these variants are the exception and not the rule. Moreover, in cases where a clinical association has been established, these relationships may not apply to different racial/ethnic populations. Further, many traits have strong developmental and environmental components and relatively low heritability. The lower part of Figure 1 shows how a single gene can result in multiple proteins, which are often expressed in different tissues or developmental stages.

In fact, most associated SNPs are not the functional SNPs, but rather in close linkage with other variants (perhaps containing the causal [functional] SNP) in the same region of the chromosome [13]. In this case, the associated SNP and the casual SNP are simply passed down together through human lineages (a phenomenon known as linkage disequilibrium) until they are separated by a relatively rare recombination event [13]. It is well recognized that different ancestry groups have varying degrees of linkage disequilibrium, and, thus, an association found in one population may not be valid for a population where it has not yet been established because the linkage between the marker SNP and the true causal variant may have been disrupted by a recombination event on the branch leading to that population [13].

### 2.3. Penetrance, Pleiotropy, Epistasis, and Polygenicity

Most traits of interest are complex, and several other genetic concepts help to explain the genotype-to-phenotype map. Penetrance is the probability of observing a trait, given that an individual has the associated variant or genotype [14]. A fully penetrant variant would be one such that everyone who had it also had the associated phenotype. It is important because in many cases having a particular genetic variant does not definitively result in the associated phenotype. Instead, it increases (or decreases) the chance of expressing that phenotype. Further, many traits are likely highly polygenic, that is, the observed phenotype is the results of contributions from many individual genes. For example, ~60 and ~100 independent loci contribute to the genetic risk associated with coronary artery disease and type 2 diabetes, respectively, with each individual locus contributing only a small effect on the underlying disease [15,16]. Some have attempted genetic risk scores (GRS) that examine the effects of multiple SNPs simultaneously; however, these scores often account for only a small proportion of the total trait variance. For example, when Vallée Marcotte et al. examined the triglyceride response to omega-3 supplementation, they found that a GRS with the top five SNPs accounted for only 11% of the total trait variance [17].

Single genes can also have multiple effects. Pleiotropy occurs when one gene is related to several different and often unrelated traits [18]. For example, sickle cell anemia is a disease caused by pleiotropy where a single gene mutation results in intense interindividual differences in the severity of the disease [19]. Epistasis occurs when the effect of one variant is dependent on the presence of other genetic variants; therefore, the full genetic architecture of the individual is important [20]. For example, SNPs in *ACE*, *FTO*, *MC4R*, and *PPARG* have all individually been associated with BMI, but, in an Italian cohort, Bordoni et al. found that the *ACE* variant appears protective against negative consequences of the *MC4R* variant [21]. In sickle cell anemia, several epistatic, pleiotropic genes (including genes that express adherence proteins, red cell receptors, and white cells) have been defined in the last decade, and many others are potential candidates [19].

In the context of nutrigenetics, polygenicity, pleiotropy, and epistasis all complicate the translation of genetic research into dietary recommendations. Phenotypic traits such as obesity, cholesterol, or plasma triglycerides have large numbers of associated variants. Consequently, it is difficult to predict what the impact of combinations, or how one variant may alter complex gene–diet interactions or other associations.

### 2.4. Genome-Wide Genetic Association Studies (GWAS)

Discerning the biological effects of the enormous catalogue of human variation is challenging. GWAS are based on the common-disease common-variant (CD-CV) hypothesis that common disease-causing alleles will underlie many common human diseases. They have played important roles in our understanding of many diseases and have identified many loci that are associated with various phenotypic traits. The basic design of GWAS studies is to take cases and controls for a given trait and to genotype several hundred thousand to a few million SNPs across the genomes of all subjects. For each SNP, a regression is performed, and a *p*-value obtained. After adjusting for multiple testing, one can then plot the *p*-values (often −log[*p*-values]) across the genome in a Manhattan plot to identify the sites that are most associated with a given trait, which appear as peaks (“skyscrapers”) in the graph. The sample sizes for a GWAS typically need to be very large—often in the 1000s—due to the very large number of SNPs that are tested.

This approach has been very popular and the GWAS catalog has grown to include over 188,000 SNP–trait associations (https://www.ebi.ac.uk/gwas/). Examples relevant to personalized nutrition include SNPs related to fasting blood glucose, macronutrient and micronutrient intake metabolism, cholesterol metabolism, obesity, vitamin D levels, and many other diet-related traits. Unfortunately, most GWAS have predominantly been performed in individuals of European ancestry, and the results found in one population do not always generalize to other populations [22]. Another key aspect of these studies is that they are typically hypothesis-generating, i.e., it is generally unknown which genes will emerge. For this reason, GWAS results should always be viewed as preliminary, in need of follow-up in additional and diverse cohorts. Even when a result has been validated in multiple cohorts, an understanding of its significance typically requires functional studies focused on how gene and protein expression as well as metabolic networks are affected.

## 3. The Anatomy of Gene–Diet Interactions

There are several components of human diets, particularly the Modern Western Diet (MWD), which, when combined with the impact of diverse genetics on the metabolism of certain nutrients, have the capacity to give rise to harmful gene–diet interactions [23]. These interactions affect the expression of metabolism-associated genes, which impact quantities or activities of enzymes that synthesize or catabolize that nutrient. Ultimately, this has the capacity to alter molecular phenotypes (levels of bioactive nutrient products and their metabolites) and clinical phenotypes including human disease.

**Figure 2:**
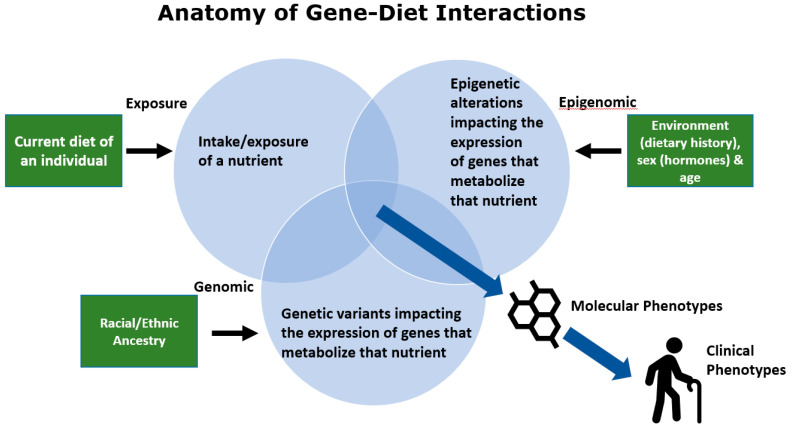
Anatomy of gene–diet interactions giving rise to molecular and clinical phenotypes.

As illustrated in Figure 2, a potentially detrimental gene–diet interaction can be impacted by several environmental, biological, and genetic components. First, gene–diet interactions can be initiated by a major change in the exposure of a particularly important nutrient to a human population. This exposure can be particularly unfavorable if the nutrient intake is altered in a genetically diverse ethnic/racial population in the absence of clinical trials to test the effect of nutrient exposure changes across all ethnic/racial groups.

An important example of this is the dramatic marked shift in fatty acid exposure which was due in large part to recommendations from health agencies beginning in 1961 in an attempt to lower serum total cholesterol and LDL lipoproteins by reducing levels of saturated fatty acids and replacing them with polyunsaturated fatty acids (PUFAs) [24] (American Heart Association, Facts on Fats, June, 2015, https://www.heart.org/idc/groups/heartpublic/@wcm/@fc/documents/downloadable/ucm_475005.pdf). Following implementation of the recommendations, food production companies began replacing saturated fatty acids, largely with omega-6 (*n*−6) 18-carbon PUFAs and particularly linoleic acid (LA). This led to a dramatic increase (~3 folds) in LA exposure while the ingestion of *n*−3 18-carbon PUFAs, such as alpha-linolenic acids (ALA), remained relatively constant [25]. This resulted in not only a dramatic increase in LA exposure but also altered ratio of dietary LA to ALA (from ~2:1 to >10:1) that enters the long-chain PUFA biosynthetic pathway. Since LA and ALA directly compete as substrates for *n*−6 and *n*−3 long-chain PUFAs, this change in exposure also altered the ratio of biologically-critical *n*−6 and *n*−3 long-chain PUFA metabolites such as *n*−6 and *n*−3 eicosanoids (prostaglandins, thromboxanes, hydroxyeicosatetraenoic acids, epoxyeicosatrienoic acids, leukotrienes, lipoxins, resolvins, protectins, and maresins) and endocannabinoids [26,27,28,29]. This change in exposure alone has been hypothesized to have created enhanced inflammation and has been accompanied by inflammation-driven diseases in certain populations and *n*−3 PUFA deficiency in others [23,30,31,32].

The second component of potentially harmful gene–diet interactions shown in Figure 2 occur when some individuals or ethnic/racial groups within a diverse population have a genetically driven, metabolically dissimilar capacity to utilize a specific nutrient than others within the same group. An example of this are variants near the *LCT* locus that code for the lactase enzyme. Lactase metabolizes lactose in milk. Cattle domestication, 5000–10,000 years ago, induced strong selection for variants in the *LCT* locus that could produce ample quantities of lactase [33]. This resulted in a high proportion of adults who could drink milk as a major carbohydrate source. However, the frequencies of these variants are dramatically different among populations with Northern European ancestry, who have two alleles for lactase persistence, and African ancestry or most Asian populations who do not have alleles for generating lactase in large quantities [33]. Clearly, alterations in nutrient exposure throughout human history have induced the selection of genetic variation to fit a wide variety of nutritional environments and consequently ancestry plays a dramatic role in the capacity of diverse populations to metabolize common nutrients. Our own work shows a similarly wide divergence in genetic variation in the fatty acid desaturase (*FADS*) locus in African, European, and Amerindian-ancestry populations, which alters the efficiency of metabolism of *n*−6 and *n*−3 dietary 18C-PUFAs [34,35,36]. Similar to lactose, this impacts the metabolism of the dietary PUFAs, and thus the interaction of genetic variation with dietary PUFA exposure can become detrimental to health in certain populations and not others.

The third component of gene–diet interactions are epigenetic alterations that influence key biological processes such as the metabolism of dietary nutrients. These epigenetic modifications change gene expression and are often heritable, but, unlike SNPs, they are not a change to the DNA sequence. One important epigenetic alteration is the methylation of DNA in and around promotor regions, which results in reduced or suppressed gene transcription and can be reversed or unmethylated. These epigenetic modifications are essential to normal biological functioning but may also be the result of environmental exposures including diet and bioactive compounds [37]. A growing body of literature has shown that both beneficial and harmful epigenetic changes can result from various dietary exposures [38]. This can include prenatal exposures; for example, children who were exposed to famine conditions in utero during the Dutch famine (1944–1945) experienced epigenetic changes in multiple genes as well as altered cholesterol and lipid profiles later in life [39,40,41].

Collectively, epigenetic changes mean that our food is not just an input to the body system but can also change how that system functions. Further, genetic variation can influence epigenetic modifications as well, adding another layer of complexity. While nutritional epigenomics is a relatively new field, it is clear that epigenetic processes play essential functional roles in how our bodies interact with food and other bioactive compounds [42]. It may also partially explain the “missing heritability” problem of GWAS, i.e., that genetic variants typically only explain a small fraction (5–10%) of a phenotype’s heritability [43].

## 4. Genetic Testing and Nutrigenetics

**Figure 3:**
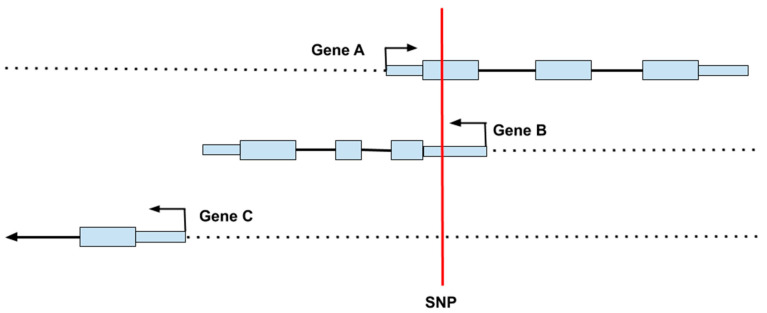
Since genes sometimes overlap, a single nucleotide polymorphism (SNP) can affect more than one gene. In this example, the SNP (shown in red) is located in the coding region of Gene A, the 5′ untranslated region of Gene B, and the upstream regulatory region of Gene C.

The most widespread, inexpensive form of genetic testing is the DNA microarray (or “SNP chip”) containing probes that can quickly detect genotypes at hundreds of thousands of SNPs across the genome [44]. Even given large numbers of genotyped and imputed SNPs, the information gained from these arrays is incomplete and contains some errors. Moreover, microarrays do not test for all types of variants and larger structural changes, such as insertions, deletions, and inversions, are not explicitly typed by this technology, although it is possible to infer gene copy numbers at known polymorphic sites from microarray data. Discerning a SNPs location is important for determining its potential functionality. Figure 3 shows an example of how a SNP can impact several different processes simultaneously, including impacting regions on more than one gene. Given the architecture of the genome, a single base change can result in alterations to multiple proteins with different functions, which might be expressed in multiple cells at different times.

A more important issue is often that rare, high-penetrance variants are unlikely to be included on an array. For practical reasons, SNPs included in array designs usually have a minor allele frequency of at least 0.5%, while mutations that disrupt genes are often present at 0.1% frequency or lower [44]. While genotypes at additional sites can often be imputed using data from large human genome databases, this is generally not the case with rare variants. Although individually rare, the probability of one rare mutation in a genome is much higher, and it is thus possible for a microarray-based test to miss the most important functional variant in an individual’s genome. Sequencing entire genomes theoretically avoids this problem, but, at present, it is still too expensive and complicated to pursue whole genome sequencing for individual nutritional purposes alone.

With these limitations in mind, a better understanding of an individual’s response to specific nutrients could help healthcare practitioners deliver more precise and effective nutrition recommendations. As mentioned above, SNPs are the simplest and most widely studied type of genetic variation; however, millions of SNPS have been identified to date [45]. Determining the functionality of individual SNPs is difficult as most SNPs have no functional effects, while others can have devastating consequences as evidenced by many rare genetic diseases. Additionally, many diet related diseases are complex with multiple genetic components and dozens of associated genetic variants. The challenge is to identify SNPs that impact diet–gene interactions and identifying those individuals and populations likely to respond to specific dietary interventions. The following examples include well studied SNPs and their effects on nutrition and health. These are summarized in Table 2.

### 4.1. Caffeine Sensitivity (CYP1A2 Gene)

A well-known example in nutrigenetics is the relationship between caffeine sensitivity and the Cytocrome P450 1A2 (*CYP1A2)* gene, which accounts for approximately 95% of caffeine metabolism and has wide interindividual variability in activity [46]. Caffeine is a naturally occurring central nervous system stimulant that is rapidly absorbed in the GI tract. Approximately 90% of adults report regular caffeine use, with an average intake of 227 mg a day, generally consumed in drinks such as coffee, tea, soft drinks, and energy drinks [47]. There is considerable variability in caffeine’s effect on humans which stems from genetic differences affecting methylxanthine, the enzyme responsible for metabolizing caffeine in the liver [47].

At least thirteen SNPs have been recognized on the *CYP1A2* gene with one, rs762551, leading to an adenine (A) to cytosine (C) allele substitution at position 163 and decreasing methylxanthine activity, which results in hypersensitivity to caffeine [48,49]. Heterozygous (A/C) carriers who have one adenine and one cystine nucleic acid and homozygous (C/C) carriers of the C allele metabolize caffeine more slowly. Homozygous carriers for the A allele (A/A) are rapid caffeine metabolizers. According to Nehlig, about 54% of the populations studied are slow caffeine metabolizers (A/C) and/or C/C carriers) while about 46% of the population are rapid metabolizers (A/A) [49]. Knowing genetic variation or polymorphism of the *CYP1A2* gene could influence caffeine intake for the general population and may affect the ergogenic effects of caffeine in athletes before exercise [50,51,52]. In one study of the diet–gene interactions of caffeine’s ergogenic effects, 35 trained male cyclists provided DNA samples and ingested 6 mg/kg of caffeine or a placebo before completing two computer-simulated 40-km time trials on a cycle ergometer. Researchers concluded that AA homozygotes had a greater reduction in time to complete 40 km (*p* < 0.05) (4.9%; caffeine = 72.4 ± 4.2 min, placebo = 76.1 ± 5.8 min) when compared to C allele carriers (1.8%; caffeine = 70.9 ± 4.3 min, placebo = 72.2 ± 4.2 min) after caffeine supplementation [50].

### 4.2. Alcohol Dependence (ADH1B Gene)

Nutrigenetics may have the potential to help in identifying individuals at risk for alcohol dependence and could help in guiding drinking behavior. Ethanol metabolism involves two enzymes, alcohol dehydrogenase and aldehyde dehydrogenase, which are encoded by seven genes (*ADH1A*, *ADH1B*, *ADH1C*, *ADH4*, *ADH5*, *ADH6*, and *ADH7*) and two genes (*ALDH1A1* and *ALDH2*), respectively [53]. The alcohol dehydrogenase 1B gene (*ADH1B*) encodes for proteins involved in the first steps of alcohol metabolism in the liver, oxidizing ethanol into acetaldehyde so that it can be eliminated from the body [53]. Thousands of rare polymorphisms have been identified in this gene; however, two are the most clinically significant: rs1229984 and rs2066702. Heterozygous (A/guanine [G]) and/or homozygous A/A carriers of the mutant allele from either SNP have increased rates of ethanol metabolism, reduced rates of alcohol consumption, and reduced risk of alcohol dependence and alcoholism [53]. This may all be due to increased levels of acetaldehyde and its associated subsequent adverse effects [53,54,55]. A variant in the rs1229984 SNP leads to the substitution of the amino acid histidine for arginine at position 48 [53]. As with many variants in these examples, this SNP is unevenly distributed among ethnic groups [53]. According to the National Institutes of Health, National Library of Medicine, the mutant variant is found in just under 5% of the population and is most common among individuals of East Asian ancestry, less common in those of Middle East ancestry, and almost absent in individuals of African and European ancestry [53,55]. The variant allele in the rs2066702 SNP which leads to the substitution of cysteine for arginine at position 370 (ADH1B*3) is found in less than 1% of the population and is more common among individuals of African ancestry [53,55].

### 4.3. Non-Alcoholic Fatty Liver Disease (PNPLA3 Gene)

Non-alcoholic fatty liver disease (NAFLD) is a major health concern with a prevalence of about 30% in Western countries and 5–18% in Asia [56]. NAFLD stems from liver accumulation of triglycerides and free fatty acids and can range in severity from hepatic steatosis (also termed non-alcoholic fatty liver [NAFL]) to non-alcoholic steatohepatitis (NASH) to the more severe cirrhosis of the liver and even hepatocellular carcinoma [56,57,58]. NAFLD is projected to become the leading driver of liver transplantation and the major cause of liver related morbidity and mortality in the next two decades [59]. The patatin-like phospholipase domain containing 3 (*PNPLA3*) gene encodes for a protein with lipase activity that acts on triglycerides in hepatocytes and retinyl esters in hepatic stellate cells. The *PNPLA3* rs738409 SNP has three genotypes, C/C, G/G, and C/G, with the G/G and C/G genotypes associated with increased risk of development of NAFLD [60] and being more common in those of Hispanic ancestry.

As a further example of the molecular underpinnings of an interaction between PNPLA3 and diet, studies have demonstrated that adults and children who are G allele carriers display an isoleucine to methionine substitution at position 148 which leads to an increase in lipogenic activity promoting triglyceride synthesis and accumulation in hepatocytes [58,61,62,63,64,65]. The *PNPLA3* gene is regulated by glucose and insulin via sterol regulatory element binding protein 1c (SREBP-1c) in mouse liver and human hepatocytes and is highly influenced by nutritional status [58,66]. Additionally, *PNPLA3* mRNA levels were shown to be influenced by both fasting (decrease) during re-feeding (increase), with elevated mRNA levels in obese, insulin-dependent mice [66]. A review by Meroni et al. suggests that PUFA supplementation, sugar restriction, and higher fruit, vegetable, and whole grain intake might help prevent NAFLD [67].

### 4.4. Obesity and Appetite (FTO Gene)

Overweight (BMI of >25) and obesity (BMI > 30) are risk factors for many chronic diseases, including type 2 diabetes, cardiovascular disease, and cancer, which affect billions of people and are a major economic burden. Fat mass and obesity-associated (*FTO*) genetic variation is associated with adiposity (BMI and waist/hip circumference), metabolic biomarkers (total cholesterol, triglycerides, and fasting glucose), and adipokines (adiponectin and leptin) [68]. The protein encoded by *FTO* is a dioxygenase enzyme which repairs alkylated deoxyribonucleic acid (DNA) and ribonucleic acid (RNA) by oxidative demethylation [68]. This protein which is highly expressed in the hypothalamus and the pituitary, both key sites for regulation of energy balance, is dependent on iron (Fe + 2) and 2-oxoglutarate (α-ketoglutarate) [69,70]. The *FTO* SNP rs9939609 has three genotypes, thymine (T)/T, A/A, and A/T. Both A/A and A/T allele carriers are predisposed to greater total body adiposity (31% higher risk) than are those with T/T alleles, partially due to altered food intake and energy expenditure [71,72,73,74]. Speakman et al. studied the diet–gene interaction with *FTO* in 150 adult participants and noted a significant increase in food (*p* = 0.024) intake with 120.7 and 294.2 more kilocalories consumed by A/A homozygous and A/T heterozygous allele carriers, respectively, than T/T types [75]. In a study of 300 children, Duicu et al. demonstrated an association with A allele carriers and obesity, elevated cholesterol, triglycerides, and adipokines [73].

In addition to increased calorie intakes, the *FTO* gene is significantly associated with increased hunger and lower satiety, possibly due to higher concentrations of serum leptin [76]. Leptin is a circulating hormone that regulates food intake and energy expenditure. It is synthesized and secreted into circulation primarily by white adipocytes and exerts its effects through a variety of central and peripheral actions [76]. Higher levels of circulating leptin have been associated with increased fat accumulation in individuals and studies show greater leptin release reduces the brain’s effectiveness in controlling hunger cues and food intake [76,77]. Labayen et al. studied 655 adolescents and found significantly higher (*p* = 0.004) frequency of A allele carriers in overweight (70.4%) vs. non-overweight (60.5%) individuals and a higher frequency of the AA genotype (*p* < 0.001) in overweight (24.1%) vs. non-overweight (14.1%) participants [78]. Additionally, the authors reported significantly higher levels of serum leptin (*p* = 0.009) in A allele carriers (22.4 ± 0.9 ng mL^−1^) than those carrying the T/T genotype (17.4 ± 1.1 ng mL^−1^) [78]. Many other studies showed similar results, and a meta-analysis by da Silva et al. showed that the TT genotype carried a significantly decreased risk of developing obesity in teens [79]. A better understanding of this key diet–gene interaction may be essential for the development of novel therapeutic approaches to address obesity.

### 4.5. Cardiovascular and Alzheimer’s Disease (APOE Gene)

Transcription of the apolipoprotein E (*APOE*) gene is critical for the production of a protein known as apolipoprotein E [80]. The *APOE* family of proteins binds lipids as part of several lipoproteins including chylomicrons, VLDL, IDL, and some HDL that are responsible for packaging and carrying cholesterol and other fats through the body. Maintaining normal levels of cholesterol is essential for the prevention of cardiovascular disease. There are three major alleles of the *APOE* gene (APOE2, APOE3, and APOE4), all of which have consistently demonstrated their roles in risk of cardiovascular disease (CVD) and Alzheimer’s disease (AD) [80,81,82,83]. The rs429358 and rs7412 SNPs together determine the *APOE* allele variant [81,82,83,84]. C/C carriers at both SNPs carry two APOE4 alleles (APOE4/APOE4) and are at >60-fold and >12-fold risk for early and late AD onset, respectively [84,85,86]. Individuals who are heterozygous carriers (C/T) at rs429358 and homozygous (C/C) carriers at rs4712 carry one APOE4 and one APOE3 allele, increasing their risk for both AD (three-fold) and heart disease (1.4-fold) [87]. More than half the population have the APOE3/APOE3 phenotype stemming from a homozygous (T/T) genotype at rs429358 and homozygous (C/C) at rs7412 [87]. Compared to these phenotypes, APOE2/APOE2 phenotypes, who carry homozygous (T/T) alleles at both SNPs, are much less likely to develop CVD and AD [85,87].

A summary of *APOE* variants and disease risk can be found in Table 3. Previous reports have shown that significant lifestyle modifications reduce CVD incidence and overall mortality [88], and those lifestyle modifications play a larger role for reducing CVD and AD incidence in APOE4 phenotype populations than in APOE2 populations [89]. Lifestyle modifications included decreasing saturated fat and cholesterol intake, increasing PUFA intake, and exercising more [88,89].

As with many of the variants discussed above, some racial/ethnic groups have a higher prevalence for specific genotypes. For example, the APOE4 isoform has the highest frequency in indigenous populations of Central Africa (29–40%), Oceania (26–49%), and Mexico (27%) with a distinct latitudinal gradient observed in Europe (5–10% in Spain, Portugal, Italy, and Greece; up to 16% in France, Belgium, and Germany; and up to 23% in the Scandinavian peninsula, with peaks of 31% in the Saami population of Finland) [90]. The APOE3 isoform shows peaks in the Alberta Hutterite people of Canada (94%), Mexican Mayas (90%), Basque and Sardinian populations (88%), and the Han Chinese (86%) [90].

### 4.6. Folate Metabolism (MTHFR Gene)

The methylenetetrahydrofolate reductase (*MTHFR)* gene encodes for the enzyme, methylenetetrahydrofolate reductase, which catalyzes the reduction of 5,10-methylenetetrahydrofolate to 5-methyltetrahydrofolate, the primary form of folate in the blood and a required element (along with vitamin B_12_) in the conversion of homocysteine to methionine [91]. There are three common SNPs giving rise to *MTHFR* alleles, with one SNP (rs1801133) well characterized as causing enzyme deficiencies [92]. The *MTHFR* SNP rs1801133 leads to an alanine-to-valine amino acid substitution in the catalytic domain of the enzyme. Heterozygous (T/C) genotype and homozygous (T/T) genotype carriers show greatly diminished *MTHFR* activity compared to homozygous (C/C) genotype carriers with T/T carriers having lower blood folate and higher blood homocysteine concentrations [93].

Ethnicity and geographic location are highly associated with the prevalence of carrying one or two minor alleles according to a study of 7130 newborns from 16 areas in the Americas, Europe, Russia, China, and Australia [94]. Specifically, 20–53% of individuals may have inherited one T copy (C/T genotype), and 3–32% of individuals may have inherited two T copies (T/T genotype) [94]. It is widely accepted that T/C and T/T genotypes at SNP rs1801133 increases total blood homocysteine levels leading to hyperhomocysteinemia, which is a risk factor for a variety of medical conditions, including adverse birth outcomes (neural tube defects, congenital heart disease, and premature delivery), pregnancy complications, cancers, adult cardiovascular diseases, and neurodegenerative disorders [95,96,97]. Supplementation of vitamin B_12_ and folic acid reduces plasma homocysteine levels by providing the substrate needed for normal metabolism. However, homocysteine-lowering treatment does not reduce the risk of cardiovascular disease in all cases with these genetic variants possibly influencing treatment outcomes [94].

### 4.7. Vitamin D Metabolism (GC Gene)

Vitamin D comes in two dietary forms: D_3_ and D_2_. D_3_ is created in the skin in response to UV light or absorbed from certain animal-based foods such as fatty fish, while D_2_ is obtained from plant-based foods [98]. Both forms are metabolized first to 25-hydroxyvitamin D (25(OH)D) by the enzyme cytochrome P450 family 2 subfamily R member 1 (CYPT2R1) and then to the active form, 1,25-hydroxyvitamin D (1,25(OH)_2_D), by cytochrome p450 family 27 subfamily B member 1 (CYP27B1) [98]. Vitamin D deficiency, often defined as a serum 25(OH)D concentration of <30 ng/mL (75 nmol/L), has long been known to cause disorders of the bone, such as rickets and osteomalacia, but has more recently been associated with an increased risk of numerous diseases including breast and colorectal cancer, CVD, and impaired immune function [99]. Genetic variants associated with blood levels of 25(OH)D have been found in many different genes, including *CYP2R1*, *CYP27B1*, and the vitamin D receptor gene vitamin D receptor (*VDR)* [100], but perhaps the best studied examples are in the gc-globulin (*GC)* gene, which codes for the vitamin D binding protein (DBP). DBP is the main carrier of vitamin D metabolites in the bloodstream where 85% of the total 25(OH)D is bound to this protein. It also may serve as a reservoir of this vital nutrient in periods when intake and synthesis are low [101].

At least 13 SNPs in the *GC* gene have been associated with circulating 25(OH)D levels [102], but two of the most studied are the missense SNPs rs4588 and rs7041, which together define the GC1s, GC1f, and GC2 haplotypes (Table 4). The rs7041 SNP results in a substitution of glutamic acid for aspartic acid at position 432, while rs4588 causes a substitution of lysine for threonine at position 436 [101]. All three haplotypes are relatively common in populations of European descent, but among Africans, GC1f is very common (>80% allele frequency) while GC2 is rare [103,104]. Many studies have found lower serum concentrations of 25(OH)D in carriers of one or more GC2 alleles with GC2/GC2 homozygotes being most at risk of deficiency (see, for example, [105,106,107]). Enlund-Cerullo et al. studied these two SNPs, along with several others, separately and in combination, in a trial of vitamin D supplementation in infants of European descent [107]. They found that the rs4588 A/A genotype and the GC2/GC2 diplotype were both associated with low serum levels of 25(OH)D at birth; these variants and the rs7041 G/G genotype were also less responsive to vitamin D supplementation [107]. Given these results, GC2 carriers (and especially GC2 homozygotes) are likely to benefit most from monitoring of serum vitamin D levels, with supplementation as needed.

### 4.8. Long-Chain Fatty Acid Biosynthesis (FADS locus)

As discussed above, dietary omega-3 (*n*−3) and omega-6 (*n*−6) PUFA metabolism helps control many essential components in human physiology including membrane fluidity, inflammation status, organ development, and much more [108]. The two essential PUFAs, *n*−3 alpha-linolenic acid (ALA, 18:3*n*−3) and *n*−6 linoleic acid (LA, 18:2*n*−6), both utilize the same two desaturase and two elongase enzymes to produce the conditionally-essential, biologically-active long-chain (LC) PUFAs eicosapentaenoic acid (EPA, 20:5*n*−3), docosahexaenoic acid (DHA, 22:6*n*−3), and arachidonic acid (ARA, 20:4*n*−6) [31]. The rate limiting steps of LC-PUFA biosynthesis are fatty acid desaturase (FADS) enzymes [109]. There are two primary FADS enzymes: FADS2 (∆6 desaturase and ∆4 desaturase) and FADS1 (∆5 desaturase). A recent study by our lab examining *FADS* genetic and metabolomic analyses has further identified the ∆5 desaturase (FADS1) step as a critical control point in the formation of biologically important lipids [109]. The FADS1 enzyme is necessary for the conversion of dihomo gamma linolenic acid (DGLA, 20:3n6) to ARA and eicosatetraenoic acid (20:4*n*−3) to EPA [31]. EPA typically undergoes one additional elongation and one additional desaturation step to become DHA [110,111]. ARA is a substrate for pro-inflammatory eicosanoids, while EPA is a substrate for mostly anti-inflammatory eicosanoids [23]. *FADS1* SNP rs174537 shows strong association between PUFA levels and has three variants: G/G, G/T, and T/T [34]. The G allele is more metabolically efficient than the T allele, allowing for greater *n*−6 and *n*−3 LC-PUFA biosynthesis [34]. When stratified by genotype, ARA and EPA show the strongest difference in plasma concentrations; ARA plasma levels are 8.13% of total fatty acids in the G/G genotype, 6.63% in the G/T genotype, and 5.39% in the T/T genotype (*p* = 1.59 × 10^−5^) [112]. Similarly, EPA levels are 0.48% in the G/G genotype, 0.36% in the GT genotype, and 0.32% in the TT genotype (*p* = 0.0024) [112].

The elevated ARA concentrations in the G/G group have been proposed to give rise to elevated inflammation associated with inflammatory diseases, while *FADS* gene–dietary PUFA interactions in the MWD may lead to *n*−3 LC-PUFA deficiency and cardiometabolic disease in T/T carriers [31,113]. Importantly, the G allele is almost fixed in African populations and the T allele is nearly fixed in indigenous American populations with European and Asian populations falling somewhere in between [36]. Low plasma and membrane concentrations of *n*−3 PUFAs have been implicated in many conditions including cardiovascular diseases [113], sepsis [114], age-related macular degeneration [115], and oligospermia [116]. Low *n*−3 LC-PUFA levels can be remedied by taking oral *n*−3 supplements daily.

## 5. Direct-to-Consumer Genetic Testing (DTC-GT)

Genetic testing can mean everything from testing a single base position to sequencing an entire genome. Commercially available products commonly used by large DTC-GT companies typically type ~600,000–700,000 sites previously identified as variable in various human populations [117]. Additionally, DTC-GT is a method of providing ancestry and health-related genetic tests directly to consumers without the involvement or supervision of a health professional. The number of companies offering tests that include personalized nutritional or dietary advice based on one’s individual genetic data has exploded in the last decade. DTC-GT companies do not provide a clinical diagnosis, but rather risk of monogenic disorders, such as intolerance and sensitivity panels (e.g., caffeine and alcohol), macronutrient and energy metabolism (e.g., NAFLD), weight management and obesity (e.g., FTO), and vitamins and mineral requirements (e.g., vitamin D metabolism), all discussed as examples earlier in this review [117].

### 5.1. Scientific Validity, Reliability, and Accuracy

Although there is some regulation of DTC-GT by the Food and Drug Administration (FDA) and the Centers for Medicare and Medicaid Services (CMS) (https://www.genome.gov/about-genomics/policy-issues/Regulation-of-Genetic-Tests), there is largely no federal oversight for most genetic tests. With that in mind, three criteria can be used to evaluate DTC-GT as a useful tool in understanding individual health risks. DTC-GT must be reliable in that the results can be reproduced, valid in that the results measure what they are claimed to measure, and accurate in that they represent a true value. Risks associated with use of DTC-GT products include both false positive and false negative findings. False positive test results indicate incorrectly that a certain genetic variant exists and may be associated with variability in DTC genetic tests. False negative findings incorrectly indicate that a certain genetic variant does not exist [118]. In a study of 49 patient samples that had identified genetic variants by DTC-GT, 40% of the variants were false-positives and some variants that designated an individual at “increased risk” were later noted to be either benign or common variants within the population when interpreted by third party testing [119]. Lastly, DTC-GT ideally should have clinical utility and provide information regarding the diagnosis, treatment, management, or prevention of a disease that will be helpful to patients (https://www.genome.gov/about-genomics/policy-issues/Regulation-of-Genetic-Tests).

### 5.2. Ethical Considerations

According to a recent review in the Journal of the American Medical Association (JAMA), DTC-GT may reduce barriers to genomic services making them more readily available to individuals who did not previously have access, including underserved and rural populations [120]. However, for non-European ancestry groups, the results may be less useful and even misleading in regard to disease risk [120]. For example, most studies using GWAS and other genetic approaches have been carried out in European and European American populations, and there is a desperate need for genetic studies in all racial/ethnic populations.

Additionally, poor health literacy and language barriers increase the potential for consumer misinterpretation of results and further widens the health-disparities gap creating ethical concerns. In 2018, Salloum et al. examined the overall awareness of genetic testing services by US rural and urban residents stratified across racial and ethnic groups [121]. Using the Health Information National Trends Survey (HINTS) from 2011 to 2014, the authors concluded that urban residents were more likely than rural residents to report awareness of DTC-GT and non-Hispanic whites were more likely to be aware of genetic testing compared with racial/ethnic minorities including Hispanic, non-Hispanic black, and non-Hispanic other [121]. To provide more accurate and useful genetic information to minority populations, additional research within these population groups is required.

Ethical considerations also include the potential for misinterpretation of results by the consumer and, in the case of some genetic variants, a negative psychological reaction to the information. A review by Marshe et al. details the results of several studies of individuals who learned they had the APOE4 allele, which is associated with a higher risk of late-onset Alzheimer’s disease [122]. Results differed across studies, but, in several, subjects reported an increase in perceived anxiety and/or depression after the higher-risk variant was disclosed. The risk of test-related distress must be balanced against the possibility of individuals being motivated to make lifestyle changes that may reduce their risk. Companies offering DTC-GT should provide consumers with both pre-test education as part of the consent process and opportunities for post-test genetic counseling to understand and make the best use of their results [123].

### 5.3. Practical Applications

Government guidelines for population-wide healthy nutrition practices were set in place in 2010 with the adoption of MyPlate (MyPlate.gov). MyPlate is a tool to set parameters for optimal food group consumption for most people in the United States. When followed, MyPlate is an underrated but powerful tool for healthy eating; however, no randomized controlled trials on MyPlate interventions have been performed and adherence to the government’s MyPlate recommendations are low. When considering precision nutrition and the impact of DTC-GT, it is important to understand if these tests will motivate individuals to make positive lifestyle changes and, therefore, be more beneficial than population-wide guidelines. For highly heritable traits and diseases, such as monogenic disorders determined by one or a few variants, genetic testing can be accurate and predictive. Alternately, when heritability is low and traits and diseases are influenced by multiple factors, the predictive capacity of single genetic variant tests will be much less accurate [124]. Due to the complexity of genetic testing, results should be interpreted by a qualified healthcare professional in the context of other factors including environment (such as dietary exposure) and personal and family medical history.

The idea that the variation in individual genes can inform an individual’s “best” diet is alluring and certainly supported by the evolutionary history of modern humans. It is powerful to imagine being provided with a personalized gene-based diet and then having a prescription to achieve optimal health. Most research suggests that only modest improvement in an individual’s diet is achieved after genetic testing, including increased fruit and vegetable consumption and decreased red meat, salt and saturated fat intake [125,126]. Some of these dietary changes are clinically significant, and DTC-GT could be considered a motivating factor to render precision nutrition more effective than generalized nutrition advice. Alternately, DTC-GT could have the opposite effect and lead individuals to “throw in the towel”. For example, when told they have an undesirable genotype (FTO) that may result in higher body weight, individuals may simply not try a weight loss plan. For genetic testing to be meaningful in the practice of nutrition counseling, there first needs to be specific measurable outcomes or goals, along with criteria for success for a particular diet. For example, if an individual is seeking to lose weight, then a certain amount of lost weight would be a logical goal. In that case, genetics may offer insight into the type of diet that might be most effective. However, in many cases (especially in the DTC-GT space), the goal is vague, such to “improve overall health” or “boost the immune system”, which makes quantitative assessment difficult. Clearly, the usefulness of genetic information in personalized nutrition will continue to evolve but this evolution will depend on an increasing understanding of gene–diet interactions and their molecular and clinical underpinnings.

As the market for DTC-GT grows, there will be a need for health professionals who can safely interpret results and relay relevant and important information in a clear and concise manner. Currently, education opportunities in nutrigenetics for health professionals include online courses, certifications from private organizations, and graduate programs in genetic counseling. Registered Dietitians (RDs), the leading authorities on nutrition in the United States, have a unique opportunity to fill the gap when it comes to nutritional genetics, although additional education would be required. RDs should be able to judge the quality of a genetic test sold directly to the public, accurately interpret results, and be able to relay to their clients that genetic testing often does not mean that a patient will go on to develop the health problem in question. Additionally, RDs should have the capacity to articulate that health risk results from DTC-GT could be false-positives, and reassuring results could be false-negatives. Most importantly, any clinical care decisions should only be made if there is confidence in the results and their interpretations [118].

## 6. Concluding Remarks

Many professional organizations have weighed in on the genetic testing debate providing position statements with information on potential benefits, potential harms, and recommendations to guide consumers [120,127,128]. In their 2014 position statement on nutritional genomics, the Academy for Nutrition and Dietetics (AND) states “The practical application of nutritional genomics for complex chronic disease is an emerging science and the use of nutrigenetic testing to provide dietary advice is not ready for routine dietetics practice” [128]. However, they go on to agree that nutritional genomics can provide insight into how diet and genotype interactions affect phenotype and contend that DTC-GT should be accompanied by access to a healthcare practitioner trained in genetic counseling for interpretation. Additionally, they state that Registered Dietitians Nutritionists need basic competencies in genetics as a foundation to understanding the complexities of nutritional genomics before incorporating this tool as part of routine dietary practice [128]. In their 2016 position statement on personalized nutrition, the International Society of Nutrigenetics/Nutrigenomics agrees that individuals have different nutrient requirements and varied metabolism; however, many aspects of genetic testing are still limited including the complexity of gene–nutrient interactions, the accuracy of genetic evaluations, and the application of genetic knowledge [127].

As pointed out in our examples above, properly performed genetic tests can clearly inform certain individuals on important dietary issues. There is also a potential positive behavioral aspect to the implementation of precision nutrition via genetic testing in that having personal genetic knowledge may help motivate constructive actions that lead to an improvement in the health of that individual. As precision nutrition emerges, it is important that health professionals, including RDs, are given education and training on how to competently use and interpret results from precision nutrition tools such as DTC-GT.

What about the question in the title of this paper: Can you eat for your genes? In general, for people who eat poorly, especially those eating a MWD, following “one-size-fits-all” population-level healthy eating guidelines such as MyPlate would help enhance their overall health in quantitative ways regardless of their genetic ancestry. However, our genetic diversity as a species was driven in part by natural selection as modern humans encountered new diets as they spread globally from Africa into Asia, Europe, and eventually the Americas [36]. As a result, dramatic differences in genotypic frequencies of many nutritionally important variants can be observed in diverse racial/ethnic populations [129]. When individuals of diverse ancestries are faced with gene–diet interactions created by uniform dietary exposures, such as the MWD, disparities often emerge in health outcomes such as the prevalence of cardiovascular disease, type 2 diabetes, and the metabolic syndrome [31]. Additionally, consideration of genetic ancestry could provide necessary information required to understand results such as racial/ethnic differences in the efficacy of omega-3 supplementation [23,36,130]. As knowledge of gene–diet interactions and other related biology increases, we expect additional examples of evolutionarily-driven population differences in responses to dietary input will emerge, such as the *FADS* case highlighted. Consequently, genomics, together with epigenomics and metabolomics, will play key roles in designing diets personalized to one’s genetic and metabolomic signatures. While current advancement is still too limited to achieve this goal, we are hopeful that in the future, research will lead the way to that reality.

## Figures and Tables

**Table 1:** Glossary of common genetic terms.

Effect Size	A measure of the size of a genetic association. Small effect sizes are common
Epistasis	When the effect of a variant depends on other genetic variants present (i.e., the genetic background)
Genotype	The two DNA bases at a given site, e.g., A/A, A/T or T/T, one from each parent
Genotype-Phenotype Map	The relationship between phenotypes and genotypes
Heritability	The degree to which a trait is transmitted across generations
INDEL	Insertion/deletion polymorphism
Linkage Disequilibrium	When nearby variants are passed down together through human lineages
Locus	A location in the genome
Penetrance	The probability of observing the associated phenotype for a given variant.
Phenotype	An observed trait, e.g., weight
Pleiotropy	When a single gene or variant controls multiple, sometimes unrelated traits
Polygenic	A phenotypic trait that is the result of small contributions from many genes
Site	A single DNA base-pair, i.e., A, C, G, or T, where the other half of the base-pair is implied
Single nucleotide polymorphism (SNP)	A site at which there are two common DNA base pairs in the population, e.g., A and T occur at 20 and 80% respectively
Variant	A DNA polymorphism, often a SNP
Causal (functional) SNP	A SNP that is responsible for the observed phenotypic association, e.g., a protein-altering mutation
Dietary exposure	The amount of a food or nutrient an individual or population consumes

**Table 2:** Summary of examples of well-studied single nucleotide polymorphisms (SNPs) and their effects on nutrition and health.

Gene	SNP	Nutrition and Health Issue	Genotype Differences		
*CYP1A2*	rs762551	Caffeine Metabolism	C/C slow metabolizer	A/C slow metabolizer	A/A rapid metabolizer
*ADH1B*	rs1229984	Alcohol Metabolism	G/G	A/G Increased ETOH metabolism	A/A Increased ETOH metabolism
	rs2066702		G/G	A/G Increased ETOH metabolism	A/A Increased ETOH metabolism
*PNPLA3*	rs738409	Non-alcoholic fatty liver disease	C/C	G/C Increased fat accumulation	G/G Increase fat accumulation
*FTO*	rs9939609	Obesity and Appetite	T/T	A/T Increased adiposity	A/A Increased adiposity
*APOE*	rs7412	Cardiovascular and Alzheimer’s Disease	T/T Lowest AD risk	C/T	C/C Increased AD risk
	rs429358		T/T Lowest AD risk	C/T	C/C Increased AD risk
*MTHFR*	rs1801133	Folate Metabolism	C/C	T/C Diminished enzyme activity	T/T Diminished enzyme activity
*GC*	rs7041	Vitamin D Transport	TT	TG	GG Lower Serum 25(OH)D
	rs4588		CC	C/A	AA Lower Serum 25(OH)D
*FADS1*	rs174537	Long-Chain Fatty Acid Biosynthesis	G/G Most efficient	T/G Varied efficiency	T/T Inefficient

Cytocrome P450 1A2 (*CYP1A2*); alcohol dehydrogenase 1B (*ADH1B*); patatin-like phospholipase domain containing 3 (*PNPLA3*); fat mass and obesity-associated (*FTO*); apolipoprotein E (*APOE*); methylenetetrahydrofolate reductase (*MTHFR)*; gc-globulin (*GC*); fatty acid desaturase (*FADS*).

**Table 3:** Apolipoprotein E (*APOE*) variants and heart disease and AD risk.

rs429358	rs7412	Genotype	Risk	Recommendation
C/C	C/C	APOE4/APOE4	Highest	Low fat, plant-based diet
C/T	C/C	APOE3/APOE4	Increased	Low fat, plant-based diet
T/T	C/C	APOE3/APOE3	Average	Plant-centered diet
T/T	C/T	APOE2/APOE3	Average	Plant-centered diet
T/T	T/T	APOE2/APOE2	Lowest	No related restrictions

AD: Alzheimer’s Disease.

**Table 4:** *GC* Haplotypes and Vitamin D Deficiency Risk.

rs7041	rs4588	Haplotype	Risk
T (432D)	C (436T)	GC1f	Average
G (432E)	C (436 T)	GC1s	Average
T (432D)	A (436K)	GC2	Highest
